# Reduction of obesity-associated white adipose tissue inflammation by rosiglitazone is associated with reduced non-alcoholic fatty liver disease in LDLr-deficient mice

**DOI:** 10.1038/srep31542

**Published:** 2016-08-22

**Authors:** Petra Mulder, Martine C. Morrison, Lars Verschuren, Wen Liang, J. Hajo van Bockel, Teake Kooistra, Peter Y. Wielinga, Robert Kleemann

**Affiliations:** 1 Department of Metabolic Health Research, Netherlands Organization for Applied Scientific Research (TNO), Zernikedreef 9, 2333 CK Leiden, The Netherlands; 2 Department of Vascular Surgery, Leiden University Medical Center, PO Box 9600, 2300 RC Leiden, The Netherlands; 3 Department of Microbiology and Systems Biology, Netherlands Organization for Applied Scientific Research (TNO), 3704 HE, Zeist, The Netherlands

## Abstract

Obesity is associated with chronic low-grade inflammation that drives the development of metabolic diseases, including non-alcoholic fatty liver disease (NAFLD). We recently showed that white adipose tissue (WAT) constitutes an important source of inflammatory factors. Hence, interventions that attenuate WAT inflammation may reduce NAFLD development. Male LDLr−/− mice were fed a high-fat diet (HFD) for 9 weeks followed by 7 weeks of HFD with or without rosiglitazone. Effects on WAT inflammation and NAFLD development were analyzed using biochemical and (immuno)histochemical techniques, combined with gene expression analyses. Nine weeks of HFD feeding induced obesity and WAT inflammation, which progressed gradually until the end of the study. Rosiglitazone fully blocked progression of WAT inflammation and activated PPARγ significantly in WAT. Rosiglitazone intervention did not activate PPARγ in liver, but improved liver histology and counteracted the expression of genes associated with severe NAFLD in humans. Rosiglitazone reduced expression of pro-inflammatory factors in WAT (TNF-α, leptin) and increased expression of adiponectin, which was reflected in plasma. Furthermore, rosiglitazone lowered circulating levels of pro-inflammatory saturated fatty acids. Together, these observations provide a rationale for the observed indirect hepatoprotective effects and suggest that WAT represents a promising therapeutic target for the treatment of obesity-associated NAFLD.

The prevalence of obesity has increased dramatically over the last 30 years and metabolic disorders associated with obesity have become a major health and economic problem worldwide^1^. Obesity is associated with a state of low-grade chronic inflammation, frequently referred to as systemic inflammation or metabolic inflammation^2^, which is thought to drive the development of several metabolic diseases including non-alcoholic fatty liver disease (NAFLD)^3^,^4^. We recently showed that adipose tissue is a critical source of inflammation in obesity and causally involved in NAFLD progression^5^. However, it is unclear whether suppression of adipose tissue inflammation would attenuate NAFLD progression.

White adipose tissue (WAT) is the primary site of energy storage. This storage function involves expansion of WAT through adipocyte hyperplasia (increase in cell number) and adipocyte hypertrophy (increase in cell size)^6^. Adipocyte hypertrophy is closely associated with WAT inflammation: in an *in vitro* experiment with isolated primary human adipocytes^7^, only very hypertrophic cells were found to secrete MCP-1, a key mediator of immune cell recruitment into WAT. Consistent with this observation, adipocyte hypertrophy is associated with infiltration of macrophages and formation of crown-like structures (CLS)^8^, a histological hallmark of inflamed WAT. Notably, a strong increase in CLS is observed at the time point at which a WAT depot has reached its maximal mass as shown very recently in a model of diet-induced obesity^5^.

It is thought that the inflamed WAT is less insulin sensitive, which enhances lipolysis of stored fat, thereby contributing to ectopic fat deposition and the development of liver steatosis^9^. In line with this, Kolak and colleagues^10^ have shown that obese patients with inflamed WAT have more liver fat than equally obese subjects without WAT inflammation. In addition to the increased fat flux, inflamed WAT may produce inflammatory factors that can contribute to systemic inflammation and promote the progression from liver steatosis to non-alcoholic steatohepatitis (NASH)^2^,^11^,^12^. However, experimental support for a causal role of WAT in the development of NASH has long been lacking. Recently we have shown that surgical removal of inflamed abdominal (epididymal) WAT in mice reduced lobular inflammation and attenuated NASH development^5^, suggesting that WAT constitutes an possible target for the treatment of NASH.

WAT inflammation may be reduced via the nuclear hormone receptor peroxisome proliferator-activated receptor-γ (PPARγ) which is predominantly expressed in adipose tissue, controlling inflammatory and metabolic processes^13^. Previous studies in humans^14^ and animals^15^,^16^,^17^, provide indication that pharmacological activators of PPARγ such as rosiglitazone may reduce the inflammatory state of WAT in obesity. We herein investigated whether rosiglitazone intervention can reduce manifest WAT inflammation and would attenuate subsequent NAFLD development. To do so, we first determined the time point at which WAT inflammation develops during high-fat diet treatment in LDLr−/− mice. Subsequently, we studied the therapeutic effect of rosiglitazone on WAT inflammation and associated NAFLD development.

## Results

### WAT inflammation starts in epididymal WAT during high-fat diet-induced obesity

**Figure 1:**
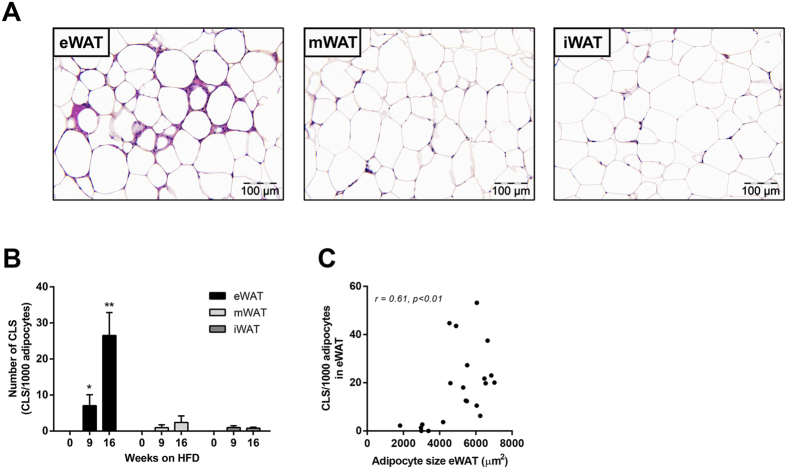
Effect of HFD feeding on development of WAT inflammation. (**A**) Representative photomicrographs of three WAT depots after 16 weeks of high-fat feeding. (**B**) Quantitative analysis of CLS formation over time in the major adipose tissue depots, eWAT, mWAT and iWAT. (**C**) Positive correlation between CLS number and adipocyte size in eWAT. Data are mean ± SEM (n =  8/group), *p < 0.05 compared with t = 0; **p < 0.05 compared with t = 0 and 9 weeks of high-fat feeding.

After 16 weeks, CLS formation was most pronounced in epididymal WAT (eWAT) (Fig. 1A), while CLS were hardly observed in mesenteric WAT (mWAT) and inguinal WAT (iWAT). Quantitative analysis showed a marked increase in CLS number in eWAT (*p* < 0.05; Fig. 1B). CLS number correlated with eWAT mass (r = 0.80, *p* < 0.001, not shown) and with average adipocyte size, a measure of adipocyte hypertrophy (r = 0.61, *p* < 0.01; Fig. 1C). The average adipocyte size in eWAT was greater than in mWAT and iWAT (not shown). Hence, eWAT is most susceptible to develop CLS, with substantial inflammation established after 9 weeks of high-fat feeding.

### Rosiglitazone attenuates WAT inflammation independent of obesity and targets WAT

Mice were treated with high-fat diet for 9 weeks to induce obesity (Table 1). At this time point, intervention with rosiglitazone was started. The caloric intake was comparable between the HFD control group and the HFD + Rosi group (14.6 ± 0.7 and 13.4 ± 0.6 kcal/day, respectively). Continuous high-fat feeding increased fasting plasma glucose, while rosiglitazone had a significant lowering effect (Table 1). Rosiglitazone also significantly lowered fasting plasma insulin and HOMA-IR relative to HFD mice (Table 1). Weight gain and total fat mass were comparable between HFD and HFD + Rosi (Table 1), indicating that the observed metabolic effects were independent of obesity.

**Figure 2:**
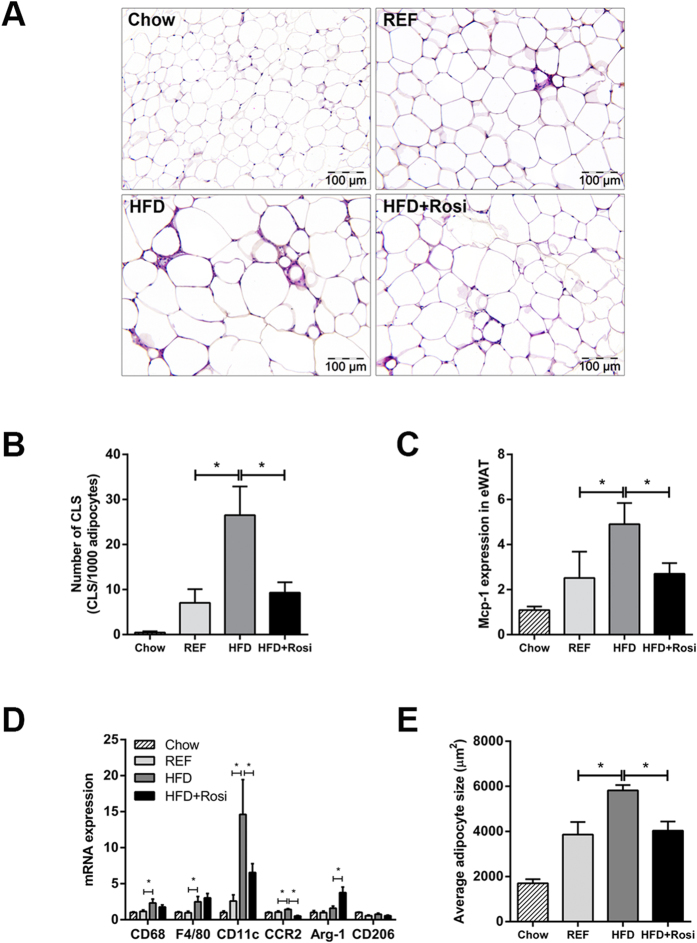
Effects of rosiglitazone intervention on eWAT inflammation. (**A**) Representative photomicrographs of HPS-stained eWAT cross-sections (magnification x200). (**B**) High-fat feeding strongly increased CLS formation in eWAT between 9 weeks (REF) and 16 weeks (HFD), while rosiglitazone fully blocked further CLS formation. (**C**) MCP-1 gene expression was increased in HFD mice, but not in HFD + Rosi. (**D**) Gene expression of macrophage markers. Rosiglitazone reduced HFD-induced expression of M1 markers (CD11c and CCR2) and increased gene expression of M2 marker Arginase-1 (Arg-1). HFD-induced expression of general macrophage markers, CD68 and F4/80, were not affected by rosiglitazone. (**E**) Morphometric analysis of average adipocyte size revealed that rosiglitazone attenuated HFD-induced increase in adipocyte size in eWAT. Data are mean ± SEM (n = 7–10/group), *p < 0.05. Mean expression of RT-PCR data was set 1 for chow-fed mice.

Quantification of CLS in eWAT revealed that CLS numbers were increased in HFD relative to REF, but remained constant in HFD + Rosi (Fig. 2A). Hence, rosiglitazone fully blocked further CLS formation but did not resolve existing inflammation (Fig. 2B). These effects were paralleled by decreased gene expression of MCP-1 in HFD + Rosi (Fig. 2C). Gene expression of macrophage markers revealed that rosiglitazone intervention reduced the pro-inflammatory M1 macrophage markers CD11c and CCR2 (Fig. 2D). In addition, rosiglitazone increased the expression of anti-inflammatory M2 macrophage marker Arginase-1, but did not affect CD206 (Fig. 2D). Consistent with this, we found less immunoreactivity against CCR2 and CD11c in adipose tissue of mice treated with rosiglitazone as determined by immunohistochemical analysis (Supplement 1). Refined analysis of CLS revealed that CLS contain CCR2 +  and CD11c +  cells and some cells expressed both markers in the HFD group as well as the HFD + Rosi group (Supplement 1). Furthermore, rosiglitazone influenced the expression of genes involved in inflammatory and oxidative stress pathways as shown by microarray analysis (Supplement 2). The observed reduction of eWAT inflammation in HFD + Rosi mice was paralleled by a decreased adipocyte size (Fig. 2E).

To validate that rosiglitazone affected PPARγ-regulated genes in eWAT under the experimental conditions employed an upstream transcriptional regulator analysis was performed. This analysis demonstrated a highly significantly increased transcriptional activity of PPARγ (Z-score: 4.1, *p* = 5.92e-24). More specifically, rosiglitazone significantly affected the expression of 1049 genes (FDR < 0.05), of which 71 are established PPARγ-regulated genes (including fatty acid transporter protein 1, fatty acid binding proteins, perilipin, uncoupling protein-1, acyl-CoA synthetase) (for detailed list, see Supplement 2). By contrast, microarray analysis of corresponding livers under the same statistical cut-off (FDR < 0.05) revealed that only 36 genes (among which 4 PPARγ-regulated genes) were differentially expressed by rosiglitazone (Supplement 3), and upstream transcriptional regulator analysis showed no activation of PPARγ. There were also no indications for off-target activation of PPARα or PPARδ from this microarray analysis (Supplement 3). Altogether, these data demonstrated that rosiglitazone significantly activated PPARγ in WAT and attenuated high-fat diet-induced WAT inflammation.

### Rosiglitazone prevents progression of NAFLD

**Figure 3:**
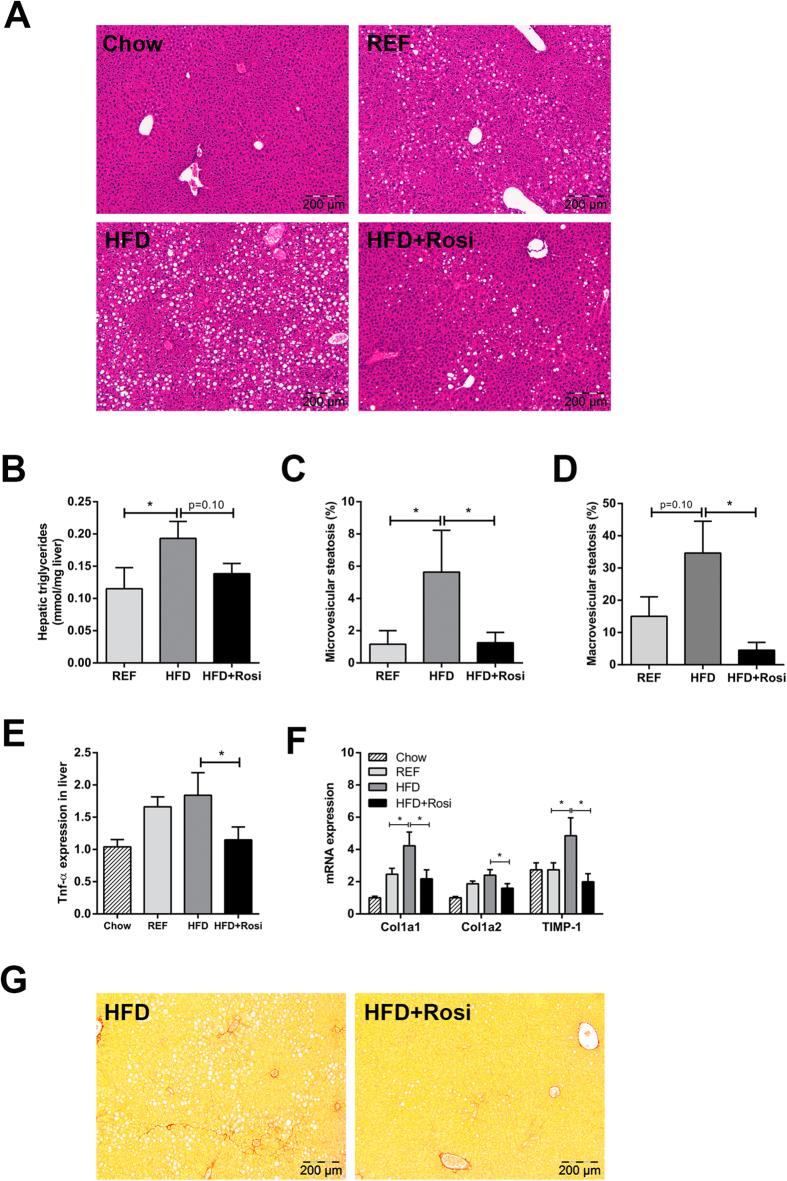
Effects of rosiglitazone intervention on NAFLD development. (**A**) Representative photomicrographs of HE-stained liver sections of REF, HFD and HFD + Rosi. (**B**) Biochemical analysis of hepatic triglyceride content. Histological quantification of (**C**) microvesicular steatosis and (**D**) macrovesicular steatosis show that steatosis was ameliorated by rosiglitazone compared with HFD (n = 7–10/group). (**E**) TNFα gene expression in liver was diminished in rosiglitazone-treated mice (n = 7–8/group). (**F**) Gene expression of fibrotic genes determined by RT-PCR. Rosiglitazone reduced HFD-induced expression of Col1a1, Col1a2 and TIMP-1. (**G**) Onset of fibrosis in Sirius Red-stained liver cross-sections in HFD mice, but not in HFD + Rosi. Pictures are shown in magnification x100. Data are mean ± SEM, *p < 0.05. Mean expression of RT-PCR data was set 1 for chow-fed mice.

Next, we investigated the effects of rosiglitazone intervention on the liver. High-fat feeding resulted in mild/moderate hepatic steatosis after 9 weeks (REF), which was markedly aggravated after 16 weeks (HFD) (Fig. 3A). Rosiglitazone blunted the progression of NAFLD and livers resembled those of REF. Biochemical intrahepatic triglyceride analysis showed a significant increase in HFD relative to REF and liver triglyceride concentrations tended to be lower in HFD + Rosi (Fig. 3B). Histological analysis revealed a strong increase in microvesicular steatosis in HFD compared with REF and rosiglitazone fully prevented this increase (Fig. 3C). Macrovesicular steatosis, a hallmark of NASH in humans^18^, was also elevated in HFD and reduced by rosiglitazone (Fig. 3D). High-fat treatment activated several pro-inflammatory and pro-fibrotic pathways in liver including those induced by TNFα (Z-score: 2.79; *p* = 3.8e-03), IL-6 (Z-score: 2.03; *p* = 2.9e-07) and TGFβ1 (Z-score: 1.3; *p* = 1.38e-05) as demonstrated by pathway analysis (FDR < 0.05). Moreover, high-fat treatment induced several genes which were recently identified in human NASH/fibrosis patients^19^ (Table 2). Rosiglitazone treatment attenuated this effect and counteracted the expression of genes including Col14a1, TaxIBP3, EFEMP2, EGFBP7, THBS2, BICC1 and DKK3. Furthermore, RT-PCR analysis of TNFα, which plays an essential role in NASH, showed increased TNFα gene expression in HFD mice and that rosiglitazone treatment quenched this induction (Fig. 3E). Similarly, HFD-induced expression of pro-fibrotic genes Col1a1, Col1a2 and TIMP-1 were suppressed by rosiglitazone intervention (Fig. 3F). High-fat feeding also resulted in infiltration of neutrophils (MPO-positive inflammatory cells) and formation of inflammatory cell aggregates characteristic for NASH^20^ between 9 and 16 weeks which was attenuated by rosiglitazone (Supplement 4). Analysis of Sirius-red stained liver cross-sections of the HFD group revealed onset of perisinusoidal fibrosis, which was not observed in HFD + Rosi (Fig. 3G). Altogether, intervention with rosiglitazone attenuated the progression from steatosis to NASH.

### Rationale for the hepatoprotective effects of rosiglitazone

**Figure 4:**
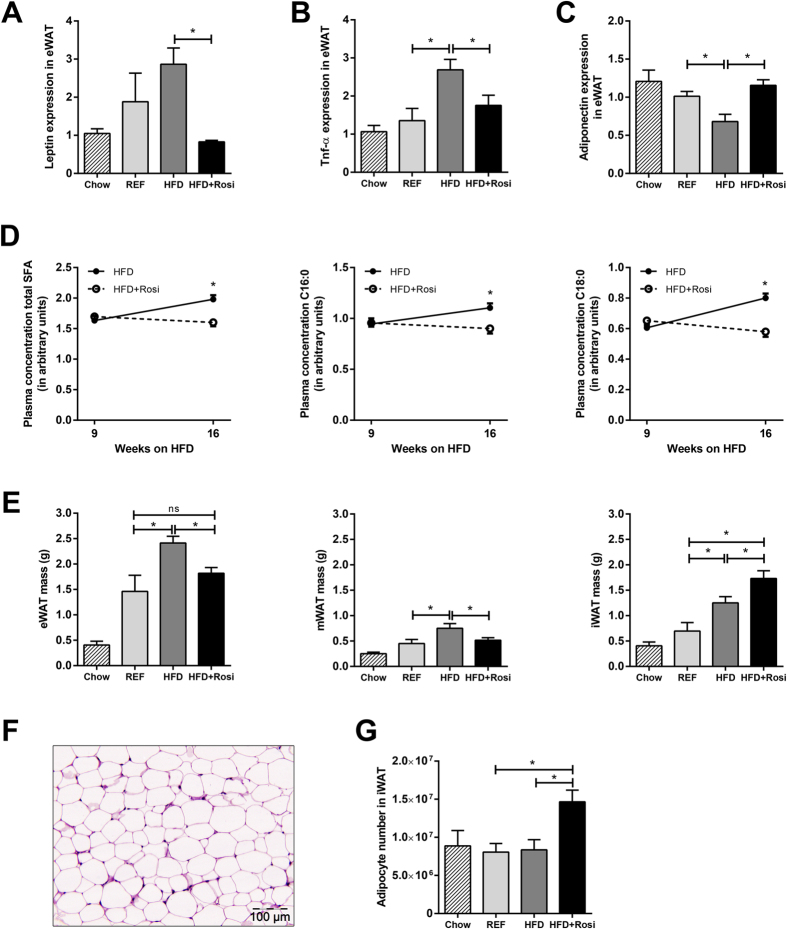
Effects of rosiglitazone on adipokine expression in eWAT, pro-inflammatory fatty acids in plasma and WAT morphology. High-fat feeding increased gene expression in eWAT of pro-inflammatory adipokines (**A**) leptin, (**B**) TNFα and decreased expression of (**C**) anti-inflammatory adipokine adiponectin, while rosiglitazone counteracted these effects. (**D**) Plasma levels of total saturated fatty acids (SFA) and specific SFAs, palmitic acid (C16:0) and stearic acid (C18:0), were increased between week 9 and 16 of high-fat feeding. This increase was blunted by rosiglitazone (all *p* < 0.01; paired t-test; n = 9–12/group). (**E**) The mass of WAT depots was increased in HFD, while rosiglitazone specifically increased iWAT mass. (**F**) Representative photomicrograph of iWAT in HFD + Rosi, showing absence of CLS. (**G**) Expansion of iWAT mass in HFD + Rosi was mainly attributable to an increase in adipocyte number. Data are mean ± SEM, *p < 0.05. Mean expression of RT-PCR data was set 1 for chow-fed mice (n = 7–8/group). Fatty acid plasma concentration was expressed as arbitrary units relative to internal standard.

In eWAT, rosiglitazone blocked the HFD-induced gene expression of leptin and TNFα (Fig. 4A,B). These effects were paralleled in plasma; HFD + Rosi reduced concentrations of leptin and TNFα (Supplement 5). By contrast, rosiglitazone fully restored the HFD-induced decrease in adiponectin gene expression in eWAT (Fig. 4C) which was also reflected in plasma (Supplement 5).

In addition, rosiglitazone prevented the high-fat diet-induced increase in total saturated fatty acids in plasma (Fig. 4D). In line with this, total NEFA were significantly increased (by 26%, *p* < 0.05) in the HFD group, whereas no significant increase was observed in the HFD + Rosi group (11%, n.s.). More specifically, plasma concentrations of palmitic acid (C16:0) and stearic acid (C18:0) were not increased in HFD + Rosi (Fig. 4D).

Since WAT inflammation correlated with WAT mass and adipocyte hypertrophy we analyzed effects of rosiglitazone on eWAT, iWAT and mWAT in more detail (Fig. 4E). During intervention with rosiglitazone, eWAT mass did not further increase while iWAT mass almost doubled, indicating a shift of fat mass from eWAT towards iWAT. Despite the increase in iWAT mass, this depot did not become inflamed (Fig. 4F). Quantification of adipocyte size showed that the expansion in iWAT was mainly attributable to an increase in adipocyte number rather than adipocyte size (Fig. 4G). This suggests that increased capability of iWAT to store fat may prevent the development of hypertrophy and associated inflammation in eWAT, and may thereby contribute to beneficial effects of rosiglitazone on NAFLD development.

## Discussion

Recent findings indicate that inflamed (abdominal) WAT plays a causal role in the development of NASH in the context of obesity^5^. WAT may thus constitute a new target for intervention. Compounds that specifically target and quench WAT inflammation have not been developed yet. We therefore used rosiglitazone, an activator of PPARγ with reported anti-inflammatory properties^14^,^15^,^16^, as a model compound to intervene in manifest WAT inflammation. Here, we show that rosiglitazone attenuates WAT inflammation and reduces NASH development.

Under the experimental conditions employed herein, rosiglitazone activated PPARγ in WAT, but not in liver, based on a comprehensive analysis of PPARγ-regulated genes. The significant activation of PPARγ in WAT may be important for the observed hepatoprotective effects, because PPARγ activation in liver could cause detrimental effects: Recent knock-out studies have shown that targeted PPARγ deletion in hepatocytes or macrophages protected mice against high-fat induced steatosis^21^, while deletion of PPARγ in adipose tissues increased liver steatosis upon high-fat feeding^22^. Furthermore, rosiglitazone treatment remained effective in mice lacking PPARγ specifically in the liver^23^, supporting the view that adipose tissue is an important site of thiazolidinedione action.

Consistent with our findings, beneficial effects of rosiglitazone in NAFLD were also observed in aged (12 months old) LDLr−/− mice that develop a more severe disease phenotype than young mice (3 months old) as used herein^24^. However, this study did not examine the effects of rosiglitazone in a therapeutic (intervention) setting and its effects in adipose tissue were not analyzed. In the study by Gupte and colleagues^24^, the diet was supplemented with cholesterol which may explain some of the differences observed on liver gene expression and inflammation. Dietary cholesterol has been shown to be a strong inducer of inflammatory gene expression in the liver^25^,^26^. For instance, treatment with a HFD supplemented with small amounts (0.2% w/w) of cholesterol triggered Kupffer cell activation and inflammatory gene expression after already 2 weeks in LDLr−/− mice, whereas the same diet without cholesterol hardly had an effect on liver inflammation^26^. High-fat diets without cholesterol supplementation induce liver inflammation typically at a slower pace and, importantly, this liver inflammation is at least partly mediated by the inflamed white adipose tissue (WAT)^5^. However, it is unclear to which extent WAT may contribute to liver inflammation when cholesterol is added to a high-fat diet.

We found that eWAT is more susceptible to develop chronic inflammation than mWAT or iWAT. This observation may be related to the fact that adipocytes in eWAT are more prone to hypertrophy than those in other adipose depots^27^. In the present study, CLS numbers in eWAT correlated with adipocyte size supporting the importance of adipocyte hypertrophy in the development of WAT inflammation^6^,^7^,^8^. Consistent with this, metabolically healthy obese subjects were found to have significantly smaller adipocytes compared with metabolically unhealthy obese patients who had more ectopic liver fat at a comparable body mass index^28^. This suggests that the ability to expand WAT through mechanisms of adipocyte hyperplasia may prevent: a) WAT inflammation and b) ectopic fat accumulation, thereby contributing to a healthy metabolic state.

We observed that rosiglitazone stimulated hyperplasia specifically in subcutaneous WAT thereby preventing adipocyte hypertrophy, which is also observed in patients treated with thiazolidinediones^29^,^30^. Consequently, this depot did not become inflamed even though its mass was much greater than in control animals, as is seen in humans treated with rosiglitazone^31^. The observed stimulation of hyperplasia specifically in iWAT by rosiglitazone may be explained by depot-specific regulation of perilipin, which is essential for enlargement of lipid droplets. Kim and co-workers showed that perilipin protein expression increased after rosiglitazone treatment in subcutaneous adipose tissue, but did not change in visceral adipose tissue^32^.

Clinical trials have shown that treatment with thiazolidinediones can improve liver histology in patients with NASH^33^,^34^. However, the underlying mechanisms mediating the beneficial effects of thiazolidinediones in NASH development are unclear. Data from the present study support the view that rosiglitazone may attenuate the development of NAFLD via an effect on WAT. Several studies showed that infiltration of macrophages into WAT is strongly associated with NAFLD development^10^,^35^,^36^. More specifically, an increase in CD11c + CD206 + and CCR2 +  macrophages in WAT is associated with enhanced production of pro-inflammatory adipokines and cytokines in WAT, and NASH severity^36^. Herein we show that rosiglitazone intervention reduced the expression of pro-inflammatory M1 markers, CD11c and CCR2 and increased the expression of anti-inflammatory M2 marker, Arginase-1. An increase in Arginase-1 expression has also been observed in HFD-fed Sv129 mice after treatment with rosiglitazone^17^, but rosiglitazone did not alter the expression of CD11c which may be related to the relatively short intervention period. Long-term rosiglitazone treatment in ob/ob mice resulted in lower CD11c expression level in WAT^37^, consisted with our findings. Analysis of CLS in the present study shows that long-term rosiglitazone intervention attenuates WAT inflammation by reducing CLS numbers (Fig. 2B), rather than altering the activation state of immune cells within a CLS (as determined CD11c and CCR2 immunoreactivity).

Our study indicates that the hepatoprotective effects on NASH by rosiglitazone may at least partly be mediated by adipokines, since plasma leptin and TNFα levels were reduced and plasma adiponectin levels were increased. It is known that leptin can exert pro-inflammatory effects and can activate hepatic stellate cells thereby promoting fibrosis^38^. TNFα plays a crucial role in human and animal NAFLD and neutralization of TNFα activity attenuated the disease^39^. For instance, adiponectin is a potent TNFα-neutralizing cytokine that counteracts inflammation that is relevant for NASH progression^38^,^39^. It has been demonstrated that also saturated fatty acids can activate inflammatory cascades leading to activation of TNFα^40^. We found that the saturated fatty acids; palmitic acid and stearic acid, were markedly increased by high-fat feeding and reduced with rosiglitazone. Notably, these fatty acids are also increased in patients with diagnosed NASH^41^. Furthermore, surgical excision of inflamed WAT in mice lowered palmitic acid in plasma and reduced progression towards NASH^5^. *In vitro* experiments have shown that conditioned medium from palmitic acid-treated hepatocytes induces the expression of pro-fibrotic genes in hepatic stellate cells^42^, providing mechanistic support for a crucial role of inflammatory lipid mediators in NASH.

We also observed that rosiglitazone attenuated the HFD-induced hepatic expression of the genes encoding for Col1a1, Col1a2 and TIMP-1. This hepatoprotective effect of rosiglitazone was further substantiated by an effect on genes that are associated with severity of human NAFLD as shown by Moylan *et al.*^19^. These findings support the view that the experimental conditions established herein (HFD-induced obesity, hyperinsulinemia, WAT inflammation concurrent with histologic NASH) may facilitate preclinical research that aims at translation to the human setting.

In all, intervention with rosiglitazone reduces WAT inflammation, lowers circulating inflammatory mediators and attenuates NAFLD progression. These effects were independent of total adiposity and body weight, indicating that adipose tissue quality (i.e. inflammatory state) rather than absolute mass is critical for NAFLD development. Our results suggest that intervention in WAT may present a new therapeutic option for the treatment of NAFLD.

## Methods

### Animal experiments

All animal experiments were approved by the institutional Animal Care and Use Committee of the Netherlands Organization of Applied Scientific Research (Zeist, The Netherlands; approval number DEC2935) and were conducted in accordance with the Dutch Law on Animal Experiments, following international guidelines on animal experimentation. Mice (aged 12–14 weeks at the start of the experiment) had ad libitum access to food and water.

#### Time-course study

Male LDLr−/− mice were fed a high-fat diet (HFD: 45 kcal% lard fat, D12451, Research Diets, New Brunswick, NJ, USA) and were sacrificed after 0, 9 and 16 weeks to collect epididymal WAT (eWAT), mesenteric WAT (mWAT) and inguinal WAT (iWAT). Tissues were prepared essentially as reported^5^.

#### Intervention study

Tissues and plasma were obtained from a large cohort study in which rosiglitazone and other interventions (e.g. fenofibrate) were analyzed^43^. Briefly, one group (n = 9) was sacrificed after 9 weeks of HFD to define the condition prior to intervention (reference, REF). The remaining mice continued on HFD (HFD, n = 13) or HFD supplemented with 0.01% w/w rosiglitazone (HFD + Rosi, n = 9, Avandia, GSK, Zeist, The Netherlands). A separate control group was kept on chow as a baseline control for microarray and RT-PCR gene expression analysis. In week 16, all animals were sacrificed and WAT depots and liver were collected. Mice (n = 2) that did not become obese after 9 weeks of high-fat feeding (i.e. body weight gain 50% less than group mean), were excluded from the analyses.

### Histological, biochemical, metabolomics and gene expression analyses

Briefly, WAT characteristics and NAFLD development were quantified histologically as described^5^,^44^. Immunohistochemistry was performed on frozen, acetone-fixed WAT sections using primary antibodies specific for CCR2 (PA5-23044, Thermo Fisher Scientific, Rockford, IL, USA) and CD11c (BD553800, BD Biosciences, San Diego, CA, USA). After incubation, biotinylated antibodies were detected by incubation with streptavidin-HRP using Nova Red as a substrate (both, Vector Laboratories, Burlingame, CA, USA). All sections were counterstained with hematoxylin. Immunopositive cells were quantified in four different cross-sections per mouse using ImageJ. Intrahepatic triglyceride concentrations were analyzed by high performance thin-layer chromatography (HPTLC)^43^. Plasma parameters were determined with commercially available assays as previously specified^43^. Plasma fatty acids were determined by gas chromatography/mass spectrometry (GC/MS)^43^. The plasma concentration of total free non-esterified fatty acids (NEFAs) was determined with NEFA-HR kit (Instruchemie, Delfzijl, The Netherlands). Illumina microarray gene expression and subsequent pathway analysis of eWAT and liver was performed following established protocols. To analyze potential off-target effects of rosiglitazone in the liver, an upstream transcriptional activator analysis was performed^45^. Microarray data were validated and confirmed by RT-PCR and changes in expression were calculated using the comparative Ct (ΔΔCt) method, expressed as fold-change relative to chow.

### Statistical analysis

All data are presented as mean ± SEM. Data were analyzed using one-way ANOVA and least significant difference (LSD) post-hoc test. Non-normally distributed data were analyzed by Kruskal-Wallis followed by Mann-Whitney U post-hoc test. Correlations were determined by Spearman’s rank correlation. Statistically significant differences in plasma fatty acids over time within HFD and HFD + Rosi were analyzed using Student’s paired *t*-test. Statistical tests were performed using Graphpad Prism software (version 6, Graphpad Software Inc., La Jolla, USA). *P* < 0.05 was considered statistically significant.

## Additional Information

**How to cite this article**: Mulder, P. *et al.* Reduction of obesity-associated white adipose tissue inflammation by rosiglitazone is associated with reduced non-alcoholic fatty liver disease in LDLr-deficient mice. *Sci. Rep.* **6**, 31542; doi: 10.1038/srep31542 (2016).

## Supplementary Material

Supplementary Information

## Figures and Tables

**Table 1:** Metabolic parameters of experimental groups.

Parameter	Chow	REF	HFD	HFD **+** Rosi
***BW gain (g)***	3.2 ± 0.5	9.1 ± 1.8*a*	17.5 ± 0.*9b*	17.1 ± 1.*2b*
***Total adiposity (g)***	1.0 ± 0.1	2.6 ± 0.6*a*	4.4 ± 0.3*b*	4.1 ± 0.2*b*
***Glucose (mM)***	11.1 ± 0.3	12.5 ± 0.7*a*	15.0 ± 0.5*b*	10.6± 0.2*c*
***Insulin (ng/ml)***	0.7 ± 0.2	2.9 ± 0.8*a*	4.65 ± 0.9*b*	1.4 ± 0.2*c*
***HOMA-IR***	8.0 ±2.4	43.1 ± 12.8*a*	78.6 ± 16.1*b*	16.3 ± 2.5*c*

Abbreviations: *Chow,* mice fed a chow diet for 16 weeks; *REF,* reference, mice receiving a HFD for 9 weeks to define condition prior to intervention; *HFD,* control mice after 16 weeks of HFD; *HFD* + *Rosi,* rosiglitazone-treated mice (intervention from 9–16 weeks). *a,* Significantly different from chow; *b,* Significantly different from chow and REF; *c,* Significantly different from HFD (all, *p* < 0.05).

**Table 2:** Microarray analysis of hepatic gene expression profile based on genes identified in human NAFLD.

Probe ID	Gene symbol	Gene name	HFD vs. Chow			HFD **+** Rosi vs. HFD		
			Fold-Change		*p*-value	Fold-Change		*p*-value
ILMN_2635229	Thbs2	thrombospondin 2	1,740	↑	9,47E-06	0,650	↓	4,62E-04
ILMN_2764588	Igfbp7	insulin-like growth factor binding protein 7	1,465	↑	1,79E-05	0,834	↓	3,52E-02
ILMN_1217309	Tax1bp3	Tax1 (human T-cell leukemia virus type I) binding protein 3	1,360	↑	7,45E-04	0,826	↓	3,28E-02
ILMN_2866901	Efemp2	epidermal growth factor-containing fibulin-like extracellular matrix protein 2	1,556	↑	7,62E-04	0,751	↓	2,70E-02
ILMN_2636424	Itgbl1	integrin, beta-like 1	1,528	↑	1,11E-03	0,905		4,33E-01
ILMN_2746556	Dkk3	dickkopf homolog 3 (Xenopus laevis)	1,402	↑	1,16E-03	0,744	↓	4,27E-03
ILMN_1258629	Col3a1	collagen, type III, alpha 1	1,887	↑	1,61E-03	0,746		1,39E-01
ILMN_2939138	Bicc1	bicaudal C homolog 1 (Drosophila)	1,460	↑	2,69E-03	0,666	↓	1,28E-03
ILMN_2746086	Tax1bp3	Tax1 (human T-cell leukemia virus type I) binding protein 3	1,334	↑	2,94E-03	0,787	↓	1,27E-02
ILMN_2980663	Aqp1	aquaporin 1	0,812	↓	7,68E-03	1,158	↑	5,74E-02
ILMN_2606210	Dpt	dermatopontin	1,459	↑	1,08E-02	0,714	↓	2,26E-02
ILMN_3007428	Sox9	SRY-box containing gene 9	0,694	↓	1,11E-02	1,245		1,23E-01
ILMN_2831656	Epha3	Eph receptor A3	1,334	↑	1,76E-02	0,901		3,84E-01
ILMN_2687872	Col1a1	collagen, type I, alpha 1	1,471	↑	3,99E-02	0,921		6,58E-01
ILMN_2747959	Dcn	decorin	1,151	↑	4,22E-02	0,874	↓	5,21E-02
ILMN_2591027	Col14a1	collagen, type XIV, alpha 1	1,176	↑	4,74E-02	0,820	↓	1,61E-02
ILMN_1223552	Fbn1	fibrillin 1	1,181		6,25E-02	0,885		1,69E-01
ILMN_1233545	Lbh	limb-bud and heart	0,782		6,40E-02	1,089		5,19E-01
ILMN_2669189	Lima1	LIM domain and actin binding 1	1,226		8,23E-02	0,956		6,98E-01
ILMN_1253806	Col1a2	collagen, type I, alpha 2	1,278		8,24E-02	0,837		2,08E-01
ILMN_2852957	Dkk3	dickkopf homolog 3 (Xenopus laevis)	1,184		8,82E-02	0,941		5,38E-01
ILMN_1214954	Cldn10	claudin 10	0,836		1,39E-01	1,143		2,68E-01
ILMN_1228374	Lima1	LIM domain and actin binding 1	1,190		1,48E-01	0,916		4,66E-01
ILMN_2980661	Aqp1	aquaporin 1	0,895		1,89E-01	1,124		1,65E-01
ILMN_1226183	Antxr1	anthrax toxin receptor 1	1,211		1,91E-01	0,815		1,63E-01
ILMN_2848305	Pnma1	paraneoplastic antigen MA1	1,144		1,96E-01	0,993		9,43E-01
ILMN_2666018	Mgp	matrix Gla protein	1,162		1,99E-01	0,914		4,39E-01
ILMN_2816180	Lbh	limb-bud and heart	1,137		2,24E-01	0,902		3,29E-01
ILMN_1257077	Jag1	jagged 1	1,160		2,33E-01	0,941		6,22E-01
ILMN_2734683	Fstl1	follistatin-like 1	1,119		2,71E-01	0,949		6,11E-01
ILMN_2596346	Dcn	decorin	1,102		3,26E-01	0,834		6,82E-02
ILMN_2597515	Ehf	ets homologous factor	1,147		3,35E-01	1,055		7,05E-01
ILMN_3001540	Lum	lumican	1,101		4,53E-01	0,817		1,17E-01
ILMN_1227817	Ank3	ankyrin 3, epithelial	1,109		4,57E-01	0,940		6,58E-01
ILMN_2769479	Lama2	laminin, alpha 2	1,116		4,87E-01	0,968		8,38E-01
ILMN_2893417	Sox4	SRY-box containing gene 4	0,929		5,57E-01	0,965		7,75E-01
ILMN_1223963	Ank3	ankyrin 3, epithelial	1,081		5,62E-01	0,835		1,83E-01
ILMN_2836637	Glt8d2	glycosyltransferase 8 domain containing 2	1,078		5,91E-01	1,229		1,45E-01
ILMN_1249021	Bcl2	B-cell leukemia/lymphoma 2	1,058		5,99E-01	0,937		5,50E-01
ILMN_1229643	Antxr1	anthrax toxin receptor 1	1,066		6,02E-01	0,779	↓	4,37E-02
ILMN_2620563	Nexn	nexilin	1,081		6,07E-01	0,771		8,88E-02
ILMN_1238000	Srpx	sushi-repeat-containing protein	1,056		6,75E-01	1,095		4,83E-01
ILMN_2621643	Col4a1	collagen, type IV, alpha 1	1,044		7,12E-01	1,125		3,12E-01
ILMN_2629486	Srpx	sushi-repeat-containing protein	0,958		7,70E-01	0,832		2,12E-01
ILMN_2686036	Tax1bp3	Tax1 (human T-cell leukemia virus type I) binding protein 3	1,030		8,04E-01	1,004		9,72E-01
ILMN_2701712	Plcxd3	phosphatidylinositol-specific phospholipase C, X domain containing 3	0,982		8,80E-01	0,943		6,22E-01
ILMN_2629804	Epha3	Eph receptor A3	0,987		9,04E-01	1,100		3,67E-01

The table lists the genes that were recently reported to be associated with NAFLD severity in humans^19^. HFD feeding of LDLr−/− mice resulted in a significant effect on 16 genes compared to chow (arrows indicate significant up- (↑) or downregulation (↓)). Rosiglitazone counteracted the effect of a HFD as shown by the comparison of HFD + Rosi vs. HFD.
