# Supplementary material for: Reduction of obesity-associated white adipose tissue inflammation by rosiglitazone is associated with reduced non-alcoholic fatty liver disease in LDLr-deficient mice
Source: Sci Rep. 2016 Aug 22;6:31542. doi: 10.1038/srep31542 (PMC4992869; doi:10.1038/srep31542)
Supplement: Supplementary Information [file srep31542-s1.pdf]

Supplementary data to:

# Reduction of obesity-associated white adipose tissue inflammation by rosiglitazone is associated with reduced non-alcoholic fatty liver disease in LDLr-deficient mice

Petra Mulder[1,2*], Martine C. Morrison[1], Lars Verschuren[3], Wen Liang[1], J. Hajo van Bockel[2], Teake Kooistra[1], Peter Y. Wielinga[1], Robert Kleemann[1,2]

[1] Department of Metabolic Health Research, Netherlands Organization for Applied Scientific Research (TNO), Zernikedreef 9, 2333 CK Leiden, The Netherlands

[2] Department of Vascular Surgery, Leiden University Medical Center, PO Box 9600, 2300 RC Leiden, The Netherlands

[3] Department of Microbiology and Systems Biology, Netherlands Organization for Applied Scientific Research (TNO), 3704 HE, Zeist, The Netherlands

***** Corresponding author, email: petra.mulder@tno.nl

## Supplement 1: Effect of rosiglitazone intervention on inflammatory CCR2-positive and CD11c-positive immune cells in adipose tissue

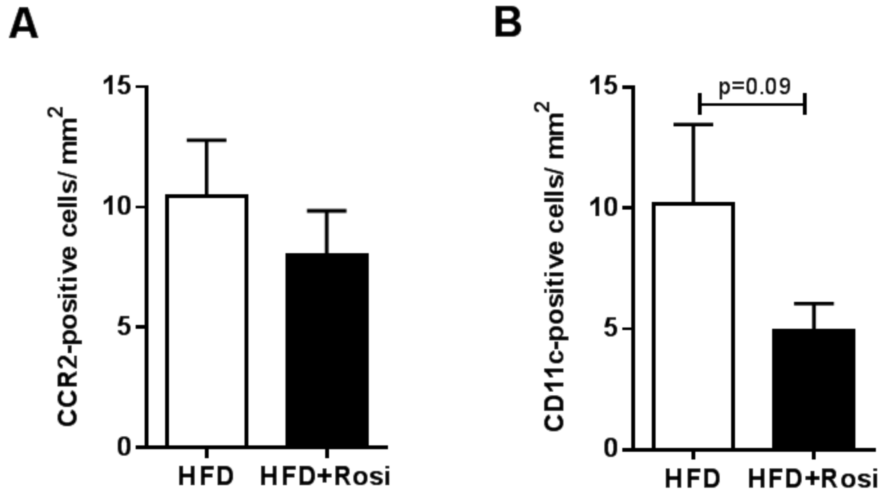


**Supplementary figure 1.** To investigate whether rosiglitazone intervention modulates adipose tissue immune cell activation, we have performed immunohistochemical stainings using primary antibodies against CCR2 and CD11c on adipose tissue cross-sections of mice fed a high-fat diet (HFD) or a HFD supplemented with rosiglitazone (HFD+Rosi). We found less immunoreactivity against CCR2 (**Supplementary figure 1A**) and CD11c (**Supplementary figure 1B**) in mice treated with rosiglitazone, which is consistent with the gene expression data. Values are expressed as mean±SEM and expressed as positively stained cells per $mm^2$ adipose tissue (n=4/group).

## Supplement 1 *continued*

**C**

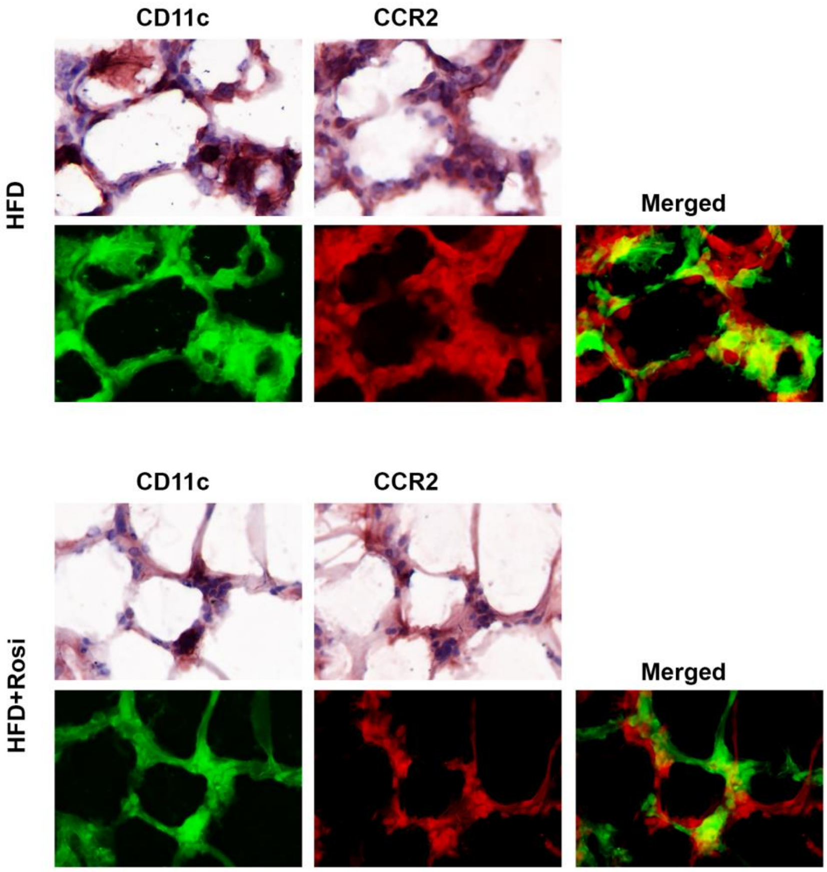


**Supplementary figure 1C** shows representative pictures of a crown-like structure (CLS) in adipose tissue of HFD-fed mice and HFD+Rosi mice, which stained positively for CCR2 and CD11c (dark brown staining, upper panels). In order to merge the coloring of the two different immunostainings in a CLS, the bright field images were converted to 8-bit immunofluorescent images (lower panels). The immunoreactivity against CD11c and CCR2 is shown in green and red, respectively. The merged images demonstrate that CLS in the HFD group and the HFD+Rosi group contain CCR2+ and CD11c+ cells and some of these cells express both markers (overlap is indicated by yellow). (Microphotographs: magnification x200).

## Supplement 2: Microarray analysis of the effects of rosiglitazone intervention in WAT

Gene set enrichment showed that rosiglitazone intervention significantly affected several biological canonical pathways related to inflammatory oxidative stress in WAT. These pathways can be grouped in three main clusters shown below and specified in **Supplementary Figure 2A-1.**

1) Oxidative phosphorylation/ Mitochondrial dysfunction
2) Oxidative stress response/peroxisomal response
3) Acute phase response/ fibrosis

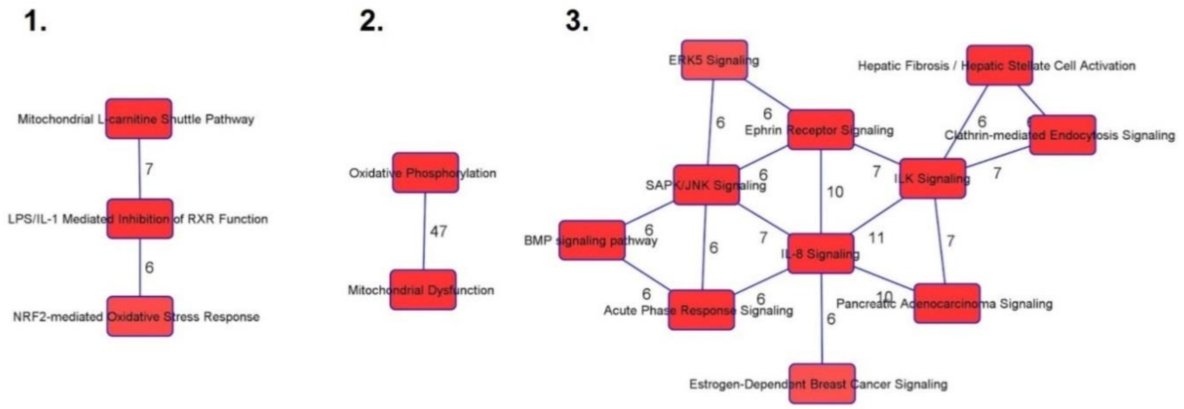


**Supplementary Figure 2A-1. Clustering of canonical pathways that are influenced by rosiglitazone intervention.** This graph shows three clusters, each of which consisting of canonical pathways. Lines between two pathways indicate that these pathways share genes (the number of overlapping genes is mentioned). The significance of the effect of rosiglitazone on individual pathways is provided in the figure below.

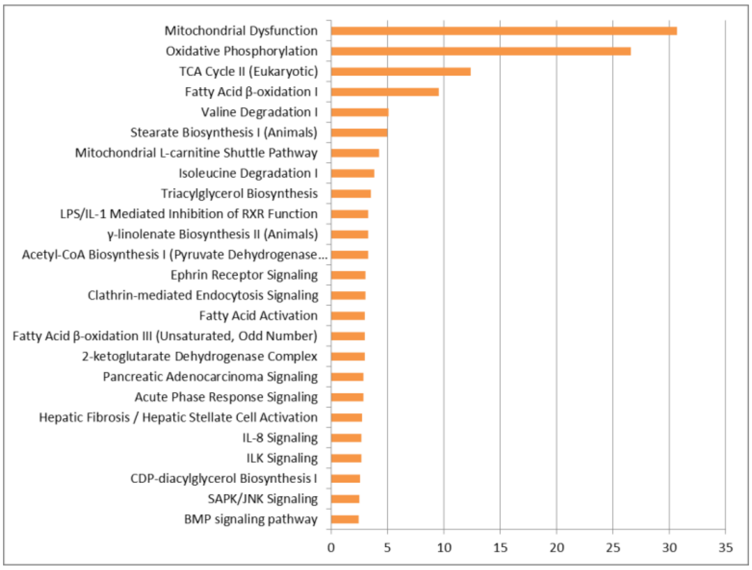


**Supplementary Figure 2A-2. Canonical pathways influenced by rosiglitazone.** The bars in the figure indicate significance of the different pathways. Significance is expressed as -log p-values.

As an example for the anti-inflammatory effect of rosiglitazone in WAT, the individual genes of the canonical pathway ‘Acute phase response signaling’ are listed below (**Supplementary Table 2A**). Among the genes that were reduced by rosiglitazone were complement factors, kinases and acute phase reactants such as haptoglobin, serum amyloid A3 and von Willebrand factor.

## Supplementary Table 2A.

| **geneSymbol** | **geneName** | **Log Ratio** | ***p*-value** |
|---|---|---|---|
| AGT | angiotensinogen (serpin peptidase inhibitor, clade A, member 8) | -2,45 | 2,50E-08 |
| C2 | complement component 2 | -1,345 | 2,69E-07 |
| C4A/C4B | complement component 4B (Chido blood group) | -1,471 | 7,47E-09 |
| CFB | complement factor B | -1,856 | 2,67E-08 |
| HP | haptoglobin | -3,09 | 4,83E-14 |
| HRAS | Harvey rat sarcoma viral oncogene homolog | 0,661 | 3,62E-03 |
| KRAS | Kirsten rat sarcoma viral oncogene homolog | 0,677 | 7,21E-06 |
| LBP | lipopolysaccharide binding protein | -2,18 | 8,91E-08 |
| MAPK12 | mitogen-activated protein kinase 12 | -0,527 | 5,78E-03 |
| MTOR | mechanistic target of rapamycin (serine/threonine kinase) | 0,466 | 1,13E-03 |
| OSMR | oncostatin M receptor | -0,7 | 4,47E-05 |
| RBP7 | retinol binding protein 7, cellular | 2,14 | 8,13E-09 |
| RELA | v-rel avian reticuloendotheliosis viral oncogene homolog A | -0,417 | 4,28E-03 |
| RIPK1 | receptor (TNFRSF)-interacting serine-threonine kinase 1 | -0,276 | 8,71E-03 |
| RRAS | related RAS viral (r-ras) oncogene homolog | -0,649 | 1,55E-03 |
| Saa3 | serum amyloid A 3 | -2,635 | 3,81E-05 |
| SERPINA3 | serpin peptidase inhibitor, clade A (alpha-1 antiproteinase, antitrypsin), member 3 | -2,923 | 3,69E-31 |
| SERPING1 | serpin peptidase inhibitor, clade G (C1 inhibitor), member 1 | -0,659 | 7,50E-04 |
| SOCS5 | suppressor of cytokine signaling 5 | 0,455 | 3,79E-03 |
| TAB1 | TGF-beta activated kinase 1/MAP3K7 binding protein 1 | -0,415 | 8,28E-03 |
| TF | transferrin | -0,933 | 1,07E-04 |
| VWF | von Willebrand factor | -1,115 | 4,49E-04 |

## Supplement 2 *continued*

**Supplementary table 2B. Differentially expressed PPARγ regulated genes in epididymal white adipose tissue affected by rosiglitazone intervention in LDLr-/- mice**. Transcriptional network analysis demonstrated a significant transcriptional activation of PPARγ (high and positive Z-score of 4.1, *p*=5.92e-24). Rosiglitazone significantly affected the transcription of 71 PPARγ regulated genes in WAT as listed below. False discovery rate of 5% (FDR<0.05) was used.

| **eWAT** | **HFD+Rosi compared with HFD** | |
|---|---|---|
| **Genes in dataset** | **Prediction (based on expression direction)** | **Log Ratio** |
| UCP1 | Activated | 3.351 |
| TSC22D3 | Activated | -0.799 |
| SORBS1 | Activated | 0.795 |
| SOD1 | Activated | 0.542 |
| SLC27A1 | Activated | 1.810 |
| SLC25A20 | Activated | 1.399 |
| SLC25A1 | Activated | 0.991 |
| PPARGC1B | Activated | 0.935 |
| PMM1 | Activated | 1.260 |
| PLIN5 | Activated | 2.068 |
| PDK4 | Activated | 3.228 |
| MGLL | Activated | 1.015 |
| ME1 | Activated | 1.048 |
| MDH1 | Activated | 0.863 |
| HP | Activated | -3.090 |
| HADHB | Activated | 1.175 |
| GPD1 | Activated | 2.167 |
| GDF15 | Activated | 1.279 |
| FABP5 | Activated | 1.361 |
| FABP3 | Activated | 2.700 |
| ESRRA | Activated | 0.490 |
| EPHX1 | Activated | -0.546 |
| EHHADH | Activated | 2.244 |
| DLAT | Activated | 1.142 |
| CYP4B1 | Activated | 1.784 |
| CS | Activated | 0.911 |
| CRAT | Activated | 1.018 |
| CPT2 | Activated | 1.249 |
| CPT1B | Activated | 3.491 |
| CIDEA | Activated | 3.514 |
| CAT | Activated | 1.016 |
| ATP5O | Activated | 1.204 |
| AQP7 | Activated | 1.205 |
| APP | Activated | -0.551 |

*Supplementary table 2B continued*

| eWAT | HFD+Rosi compared with HFD | |
|---|---|---|
| **Genes in dataset** | **Prediction (based on expression direction)** | **Log Ratio** |
| ADIG | Activated | 0.902 |
| ACTA2 | Activated | -1.767 |
| ACSL1 | Activated | 1.200 |
| ACOX1 | Activated | 1.222 |
| ACADS | Activated | 1.566 |
| ACADM | Activated | 1.035 |
| ACAA2 | Activated | 1.116 |
| Acaa1b | Activated | 1.927 |
| VEGFA | Inhibited | -1.435 |
| PRODH | Inhibited | -1.418 |
| PPIC | Inhibited | -0.959 |
| PPARGC1A | Inhibited | 0.934 |
| MLYCD | Inhibited | 1.078 |
| LAMB3 | Inhibited | -1.329 |
| GATA2 | Inhibited | -0.889 |
| ELOVL6 | Inhibited | -1.391 |
| CFD | Inhibited | -1.274 |
| CCND1 | Inhibited | -0.791 |
| APOE | Inhibited | -0.737 |
| ACSL5 | Inhibited | -0.576 |
| Abcb1b | Inhibited | -0.826 |
| ACADL | Affected | 1.571 |
| ACOT8 | Affected | 0.575 |
| AGT | Affected | -2.450 |
| CDK2 | Affected | -0.597 |
| FBP2 | Affected | 1.359 |
| HR | Affected | -1.589 |
| HSD3B7 | Affected | -0.842 |
| IGFBP3 | Affected | -1.141 |
| IGFBP5 | Affected | -1.650 |
| IGFBP7 | Affected | -0.404 |
| PDHB | Affected | 0.850 |
| S100A8 | Affected | -2.150 |
| SCNN1G | Affected | 1.209 |
| SCP2 | Affected | 1.216 |
| UGT1A9 (includes others) | Affected | -1.200 |
| VLDLR | Affected | 0.723 |

## Supplement 3: Rosiglitazone intervention does not activate PPAR-regulated genes in livers of LDLr-/- mice

Original transcriptomics data: http://www.ebi.ac.uk/arrayexpress/experiments/E-MTAB-1063/

Supplement 3A: Table listing 36 genes that are differentially expressed by rosiglitazone in liver.

| probeID | geneSymbol | geneName | Log Ratio | Adjusted p-value |
|---|---|---|---|---|
| ILMN_2925947 | ABAT | 4-aminobutyrate aminotransferase | 0,505 | 4,05E-02 |
| ILMN_2629112 | ACER2 | alkaline ceramidase 2 | 0,68 | 5,03E-03 |
| ILMN_2965414 | ANKRD22 | ankyrin repeat domain 22 | 0,94 | 4,83E-06 |
| ILMN_2834123 | APOA4 | apolipoprotein A-IV | -2,376 | 4,48E-03 |
| ILMN_2641301 | APOA5 | apolipoprotein A-V | -0,823 | 9,36E-03 |
| ILMN_2732601 | ARSG | arylsulfatase G | -0,745 | 9,36E-03 |
| ILMN_2776603 | Ccl9 | chemokine (C-C motif) ligand 9 | 0,621 | 1,75E-02 |
| ILMN_2835423 | CFD | complement factor D (adipsin) | 4,666 | 1,33E-06 |
| ILMN_2609813 | CHI3L1 | chitinase 3-like 1 (cartilage glycoprotein-39) | 1,455 | 1,88E-02 |
| ILMN_1215446 | CIDEA | cell death-inducing DFFA-like effector a | 3,387 | 1,99E-03 |
| ILMN_2827217 | CLSTN3 | calsyntenin 3 | 1,349 | 1,03E-02 |
| ILMN_2806996 | CREB3L3 | cAMP responsive element binding protein 3-like 3 | -0,52 | 3,19E-03 |
| ILMN_2960325 | CTSE | cathepsin E | 0,926 | 3,24E-02 |
| ILMN_2664224 | EPHX1 | epoxide hydrolase 1, microsomal (xenobiotic) | -0,657 | 4,60E-02 |
| ILMN_2710698 | FGF21 | fibroblast growth factor 21 | -1,51 | 2,59E-02 |
| ILMN_2605941 | GNPAT | glyceronephosphate O-acyltransferase | 0,389 | 1,67E-02 |
| ILMN_3007956 | GZF1 | GDNF-inducible zinc finger protein 1 | 0,398 | 4,72E-02 |
| ILMN_2795520 | HLA-A | major histocompatibility complex, class I, A | -0,523 | 2,71E-02 |
| ILMN_2662160 | IMPA2 | inositol(myo)-1(or 4)-monophosphatase 2 | -0,779 | 4,48E-03 |
| ILMN_1229605 | INHBE | inhibin, beta E | -0,918 | 3,24E-02 |
| ILMN_2692723 | LPL | lipoprotein lipase | 1,261 | 2,51E-04 |
| ILMN_1225764 | MFSD2A | major facilitator superfamily domain containing 2A | -1,977 | 2,58E-02 |
| ILMN_2813830 | NT5E | 5'-nucleotidase, ecto (CD73) | 0,992 | 3,69E-02 |
| ILMN_2680628 | Pbld2 | phenazine biosynthesis-like protein domain containing 2 | -0,442 | 3,20E-02 |
| ILMN_2924754 | PDZK1 | PDZ domain containing 1 | -0,315 | 7,40E-03 |
| ILMN_1249694 | PGM3 | phosphoglucomutase 3 | -0,536 | 3,20E-02 |
| ILMN_2739760 | PRELP | proline/arginine-rich end leucine-rich repeat protein | -0,429 | 1,88E-02 |
| ILMN_1254902 | RDH16 | retinol dehydrogenase 16 (all-trans) | 1,318 | 1,33E-06 |
| ILMN_1240471 | RETSAT | retinol saturase (all-trans-retinol 13,14-reductase) | -0,719 | 1,88E-02 |
| ILMN_2825446 | SDCBP2 | syndecan binding protein (syntenin) 2 | 1,575 | 3,19E-03 |
| ILMN_2684145 | SDCBP2 | syndecan binding protein (syntenin) 2 | 0,999 | 7,40E-03 |
| ILMN_2826264 | SERINC2 | serine incorporator 2 | -0,72 | 1,88E-02 |
| ILMN_1231573 | SERPINB1 | serpin peptidase inhibitor, clade B (ovalbumin), member 1 | 1,994 | 4,83E-06 |
| ILMN_1256644 | SLC6A12 | solute carrier family 6 (neurotransmitter transporter), member 12 | 0,482 | 2,58E-02 |
| ILMN_2695199 | ST3GAL6 | ST3 beta-galactoside alpha-2,3-sialyltransferase 6 | 0,826 | 5,02E-05 |
| ILMN_2757599 | TSPAN31 | tetraspanin 31 | -0,415 | 5,62E-04 |
| ILMN_1236758 | WFDC2 | WAP four-disulfide core domain 2 | -2,028 | 1,18E-03 |

Supplement 3B: Specific analysis of PPARγ-regulated genes in liver

Only 4 out of the 36 differentially expressed genes in the liver can potentially be regulated by PPARγ. These 4 genes are listed below. However, also other transcriptional regulators control the expression of these genes and a composite analysis of all gene expression changes (see upstream transcriptional regulator analysis below) shows that PPARγ is not activated.

| **Liver** | **HFD+Rosi compared with HFD control** | |
|---|---|---|
| **Genes in dataset** | **Prediction (based on expression direction)** | **Log Ratio** |
| CFD | Activated | 4.666 |
| CIDEA | Activated | 3.387 |
| LPL | Activated | 1.276 |
| EPHX1 | Activated | -0.657 |

Supplement 3C: Upstream transcriptional regulator analysis for PPARγ, PPARα and PPARδ in liver

Transcriptional network analysis of differentially expressed genes in liver demonstrated that there is no significant transcriptional activation of upstream regulators PPARγ, PPARα or PPARδ by rosiglitazone. False discovery rate of 5% (FDR<0.05) was used.

As reference for the validity of the method and for PPARα activation, we have used livers from LDLr-/- mice treated with fenofibrate (0.05% w/w), a pharmacological ligand of PPARα. Diets and duration of intervention were the same as for rosiglitazone. Fenofibrate significantly activates PPARα activity (Z-score: 7.9, $p$ =4.5e-55) based on gene expression changes induced by fenofibrate. Consistent with this, a significant activation of several key processes of lipid metabolism in liver was observed with fenofibrate (see **Supplementary Table 3C**). These processes were not affected with rosiglitazone treatment. In line with this, hepatic concentrations of β-hydroxybutyrate (beta-oxidation product) were increased with fenofibrate treatment, but not with rosiglitazone (**Supplementary Figure 3C**), further supporting absence of PPARα activation by rosiglitazone.

**Supplementary Table 3C**

| **Top bio functions in lipid metabolism** | **Predicted Activation State** | **Log ratio** | ***p*-value** |
|---|---|---|---|
| transport of long chain fatty acid | Increased | 2,902 | 1,91E-09 |
| oxidation of fatty acid | Increased | 2,782 | 1,04E-17 |
| uptake of long chain fatty acid | Increased | 2,634 | 1,20E-03 |
| storage of lipid | Increased | 2,58 | 3,39E-06 |

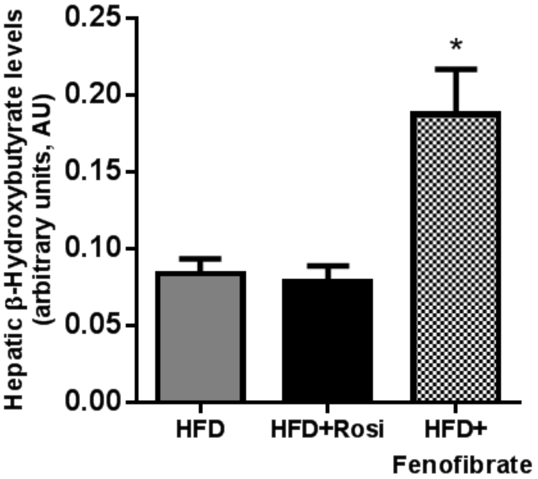


**Supplementary figure 3C** Using liver tissue homogenates and GC-MS technology, we examined whether rosiglitazone would affect hepatic β-hydroxybutyrate levels (a marker of hepatic fatty acid oxidation) relative to HFD mice. As a positive control for activation of PPARα, livers of fenofibrate-treated LDLr-/- mice were used. The levels of hepatic β-hydroxybutyrate were not increased by rosiglitazone, but were significantly increased by fenofibrate. These data further support absence of PPARα activation by rosiglitazone. Values are expressed as mean ± SEM and expressed as arbitrary units (AU) relative to internal standard. *$p<0.05$ vs. HFD, HFD+Rosi.

## Supplement 4: Effect of rosiglitazone on myeloperoxidase (MPO)-positive cells (neutrophils) in liver

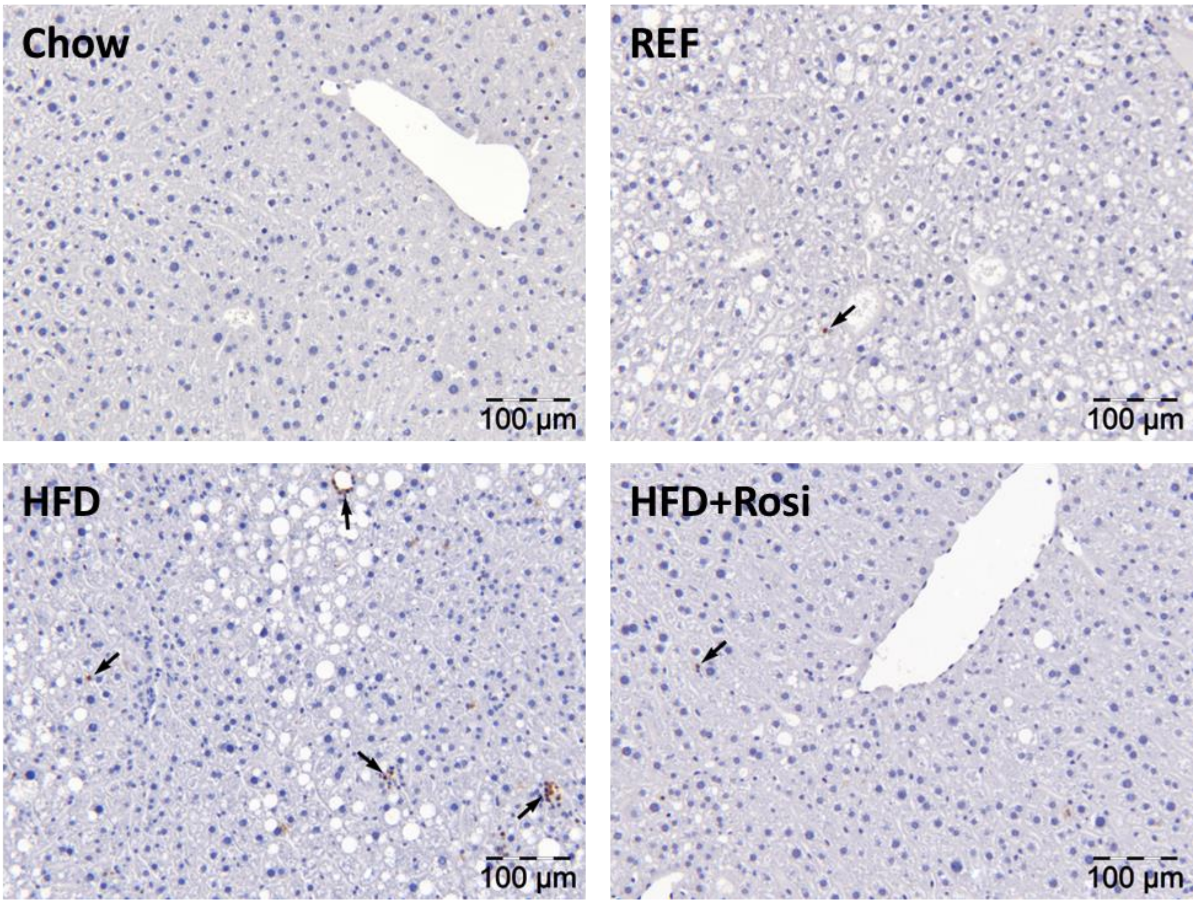


Representative pictures of MPO immunohistochemical stained liver sections of mice after a chow diet, 9 or 16 weeks high-fat diet (REF and HFD, respectively) or rosiglitazone intervention (HFD+Rosi). Infiltration of neutrophils into the liver was observed after 16 weeks of HFD (both single cells and cell aggregates). Only a few MPO-positive cells were observed in HFD+Rosi, which resembled the condition prior to intervention (REF). (magnification x100).

## Supplement 5: Effect of rosiglitazone on adipokine plasma concentrations of leptin, TNFα and adiponectin

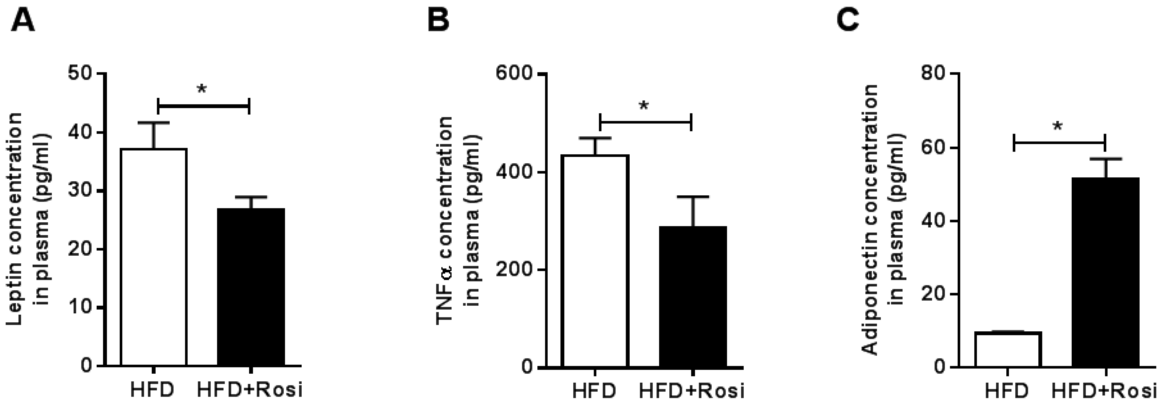


Plasma concentrations of (A) leptin, (B) TNFα, and (C) adiponectin of mice fed either a high-fat diet (HFD) or a HFD supplemented with rosiglitazone (HFD+Rosi). Rosiglitazone intervention reduced levels of pro-inflammatory adipokines leptin and TNFα. By contrast, rosiglitazone increased levels of anti-inflammatory adipokine adiponectin. Values are expressed as mean±SEM and expressed as plasma concentrations in pg/ml. *$p<0.05$.
